# The efficacy and safety of Kyung-Ok-Ko on cancer-related fatigue in lung cancer patients: Study protocol for a randomized, patients-assessor blind, placebo-controlled, parallel-group, single-center trial

**DOI:** 10.1097/MD.0000000000017717

**Published:** 2019-11-01

**Authors:** Kwan-Il Kim, Moonkyo Kong, Seung Hyeun Lee, Beom-Joon Lee

**Affiliations:** a Division of Allergy, Immune and Respiratory System, Department of Internal Medicine, College of Korean Medicine, Kyung Hee University; b Department of Clinical Korean Medicine, Graduate School, Kyung Hee University; c Division of Lung and Head and Neck Oncology, Department of Radiation Oncology, Kyung Hee University Medical Center, Kyung Hee University School of Medicine; d Division of Pulmonary and Critical Care Medicine, Department of Internal Medicine, Kyung Hee University Medical Center, Kyung Hee University School of Medicine, Dongdaemun-gu, Seoul, Republic of Korea.

**Keywords:** cancer-related fatigue, herbal medicine, Kyung-Ok-Ko, lung neoplasm

## Abstract

**Background:**

Cancer-related fatigue (CRF) is a major symptom experienced by lung cancer patients receiving chemotherapy and radiation therapy. Since CRF has a multidimensional influence on cancer patients, they may experience physical weakening, a decline in cognitive function, and depression from emotional consequences. Kyung-Ok-Ko is used for improving fatigue or weak physical constitution. It is known to be effective in immune activation, reducing fatigue, and enhancing cognitive function. Although Kyung-Ok-Ko is clinically used for the treatment of CRF, its efficacy and safety against CRF in lung cancer patients are yet to be studied. Therefore, we aimed to investigate the efficacy and safety of Kyung-Ok-Ko.

**Methods:**

This is a randomized, placebo-controlled, patients-assessor blind, parallel-group, single-center clinical trial. Lung cancer patients with CRF, after termination of chemo or radiation therapies, are randomized in a 1:1 ratio to receive either Kyung-Ok-Ko or placebo for 6 weeks. The primary outcome is Brief Fatigue Inventory (BFI). The secondary outcomes include Visual Analog Fatigue Scale (VAFS), Functional Assessment of Cancer Therapy (FACIT) Fatigue scale, Hospital Anxiety Depression Scale (HADS), Montreal Cognitive Assessment Korean version (MoCA-K), and Korean pattern identification questionnaire. Adverse events are evaluated by Common Terminology Criteria for Adverse Events (CTCAE). All outcomes and adverse events are assessed at the baseline, mid-treatment, post-treatment, and at 1-month follow-up.

**Discussion:**

This study investigates whether Kyung-Ok-Ko can alleviate CRF in lung cancer patients. The results of this study will provide clinical evidence for the application of Kyung-Ok-Ko in the treatment of CRF in lung cancer patients.

**Trial registration:**

Korean Clinical Trial Registry (http://cris.nih.go.kr; registration number: KCT000666).

Trial status: Currently, participant recruitment is ongoing.

## 1 Introduction

Lung cancer is the leading cause of deaths due to cancer in both men and women worldwide.^[1]^ In the United States, 0.25 million new cases of lung cancer along with the highest mortality rate was reported in 2018.^[2]^ Lung cancer treatments involve multimodality approaches, including surgery, radiotherapy, and cytotoxic chemotherapy, or molecularly targeted agents. During or post-treatment, a number of cluster symptoms, such as anxiety, fatigue, and dyspnea, appear.^[3]^ About 60% to 96% of patients who receive anticancer treatments complain of fatigue.^[4]^

Cancer-related fatigue (CRF) has been referred to by a panel of the National Comprehensive Cancer Network (NCCN) as a distressing, persistent, subjective sense of physical, emotional, and/or cognitive tiredness or exhaustion related to cancer or cancer treatment that is not proportional to recent activity and interferes with usual functioning.^[5]^ Since the influence of CRF on cancer patients is multidimensional, they may experience physical weakening, a decline in cognitive function due to deteriorating mental strength, and depression from emotional consequences.^[6]^

CRF is experienced more by lung cancer patients compared to other types of cancer, and many patients were reported to experience CRF with disabilities.^[7]^ It decreases the patient's quality of life, resulting in reduced compliance with chemotherapy, and ultimately has a negative effect on the survival of the patient; it is an index for the decreasing survival rate of cancer patients.^[8,9]^ An 8-year long cohort study, comprising 2405 lung cancer patients, was conducted and among the symptoms of fatigue, coughing, and dyspnea that the patients frequently complained of, only fatigue was identified as a significant predictor of the poor 4-year and 5-year survival rate.^[10]^ In addition, according to a recently reported study, not only improving the quality of life but also self-evaluation of the symptoms affecting the quality of life alone was shown to increase the survival rate in patients receiving cancer treatment.^[11]^

The major reasons for CRF are the side effects of cancer treatment. Although the exact mechanism is not known, all treatments, including chemo and radiation therapy, molecular targeted agents, and surgical treatment can induce CRF.^[5]^ Although CRF is induced by various factors, many studies have reported inflammation as the common factor of the mechanism.

Since CRF is the main reason for the interruption in treatment or exacerbation of the disease, much effort has been focused on the management of CRF. However, it remains underdiagnosed and undertreated, without any standard therapy until now.

Kyung-Ok-Ko is a prescription, which is described in Donguibogam, a traditional Korean medicine book, and consists of Ginseng Radix, Poria Sclerotium, Rehmanniae Radix Crudus, and honey. It has been widely used for improving fatigue or weak condition. It has been developed as a traditional Korean medicine based on national standards approved by the MFDS (Ministry of Food and Drug Safety). It is reported via in *vitro* and clinical studies that Kyung-Ok-Ko activates the immune system,^[12]^ reduces fatigue,^[13,14]^ and enhances the cognitive function.^[15]^ Anti-inflammatory effects have also been reported, as a supporting mechanism.^[16–18]^

Although Kyung-Ok-Ko is used for alleviating fatigue, research on CRF in lung cancer patients is limited. Therefore, we aimed to identify the efficacy and safety of Kyung-Ok-Ko on lung cancer patients experiencing CRF.

## 2 Methods

### 2.1 Objective and design

**Figure 1:**
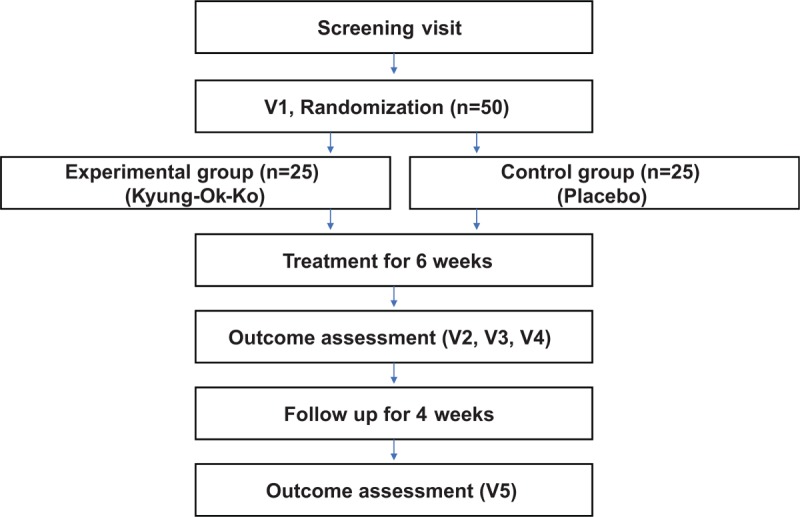
Study flowchart.

This is a randomized, placebo-controlled, patients-assessor blind, parallel-group and single-center clinical trial. This study was designed to evaluate the efficacy and safety of Kyung-Ok-Ko in lung cancer patients who have received cancer treatment and are experiencing CRF. The trial will be conducted for a total of 10 weeks, as patients who experienced CRF 2 weeks after completing cancer treatment will take the clinical trial drug for 6 weeks, followed by a 4-week observation period. A flow diagram of the study is presented in Figure 1.

### 2.2 Randomization, allocation, and blinding

Subjects are randomized in a 1:1 ratio to the experimental group (Kyung-Ok-Ko group) and control group (placebo group). For randomization, a statistician independent of this clinical trial will use SAS ver.9.1.3 (SAS Institute Inc., Cary, NC) for Microsoft Windows to randomly assign numbers to create a randomization table. The experimental group or placebo group assignments according to the table of random numbers and random numbers generated using SAS are kept by a third party until termination or completion of the trial, and investigators do not require them. The study drug and placebo are assigned and managed by a code and a patients-assessor blind method, where the investigators and subjects cannot distinguish study drug and placebo, was followed. For this purpose, the placebo was manufactured similar to the study drug in terms of appearance, flavor, and scent. The manufacturing company conducted code labeling assigned for the study drug and placebo, and a third party matches the random number and code. When registering each study subject, the third party provided the management number of the study drug or placebo that has been matched with the random number to the investigator via telephone, text message, or mobile communication application. Clinical trial drug and placebo were kept in the pharmacy of Kyung Hee University Korean Medicine Hospital Clinical Trial Center (K-CTC) and was delivered to the subjects by a pharmacist who was kept blind. If the blinding should be broken prior to completion of the clinical trial due to serious adverse events (SAEs) during the clinical trial period, the reasons for breaking blindness will be reviewed and approved before breaking the blindness by the principal investigator.

### 2.3 Patients

#### 2.3.1 Inclusion criteria

(1) Men and women aged above 19 years.
(2) Lung cancer patients who received cancer treatment (chemo and radiation therapy).
(3) Patients who have completed cancer treatment >2 weeks ago and do not have any further cancer treatment plans.
(4) Patients experiencing fatigue, with brief fatigue inventory (BFI) ≥4.
(5) Patients with Eastern Cooperative Oncology Group (ECOG) scale ≤2.
(6) Patients who voluntarily decided to participate in this clinical trial and signed the consent form.

#### 2.3.2 Exclusion criteria

(1) Patients suspected of relapse or progression according to chest CT or chest X-ray.
(2) Patients with hemoglobin level <9 g/dL, or platelets <50 000/mL, or absolute neutrophil count <1000/mL.
(3) Patients with uncontrolled thyroid disorder (free thyroxine >1.79 ng/dL, thyroid stimulating hormone >10 μm/dL).
(4) Patients with moderate liver and kidney impairment (increase in aspartate transaminase (AST), alanine transaminase (ALT) by >3 times, or increase in creatinine by >2 times).
(5) Patients who do not expect >6 months of life expectancy after cancer diagnosis.
(6) Patients diagnosed and treated for severe dementia (Montreal Cognitive Assessment Korean version, MoCA-K <10), severe depression (Hospital Anxiety Depression Scale, HADS >15).
(7) Patients who are deemed difficult for clinical trial performance.
(8) Early-stage lung cancer patients who are treated with surgery alone and late-stage lung cancer patients who are not receiving cancer treatment.
(9) Patients who received oriental medicine or pharmacotherapy (methylphenidate, modafinil, bupropion) for the purpose of improving CRF in the last 4 weeks.
(10) Patients who are pregnant or breastfeeding.
(11) Patients who did not agree to contraception during the clinical trial period.
(12) Patients who participated in a clinical trial for the same disease, in the last 3 months.
(13) Patients who are not eligible to participate in this trial at the discretion of the investigator.

#### 2.3.3 Withdrawal criteria

(1) Subjects who received treatment that could affect the outcome without the investigator's instructions during the study period.
(2) Subjects who do not follow clinical study protocol or have compliance <80%.
(3) Subjects who cannot continue clinical trial due to adverse events (AEs) or if serious AEs occurred.
(4) Subjects who withdraw consent.
(5) Subjects who use methylphenidate, modafinil, bupropion, and oriental medicines during clinical trial, which can influence fatigue improvement.
(6) Subjects who are determined by the investigator to be ineligible for participation in the clinical trial.

### 2.4 Interventions

#### 2.4.1 Experimental group

The experimental group is administered Kyung-Ok-Ko, which is a dark-brown paste preparation (23.5 g per package), twice a day for a total of 6 weeks. Kyung-Ok-Ko is manufactured by Kwang Dong Pharmaceutical Co., Ltd (Seoul, Korea), which is approved by the MFDS in accordance with the Korean Good Clinical Practice (GCP) regulations. The 23.5 g Kyung-Ok-Ko is composed of 7.98 g of Rehmanniae Radix Crudus, 2.48 g of Poria Sclerotium, 1.24 g of Ginseng Radix, and 8.3 g of honey and other excipients. The subjects receive a 14-day package per visit.

#### 2.4.2 Placebo group

The placebo group is administered placebo Kyung-Ok-Ko (23.5 g per package) twice a day for a total of 6 weeks. The placebo is manufactured by Kwang Dong Pharmaceutical Co., Ltd, similar to the physical traits of the clinical trial drug. The placebo that is very similar in size, flavor, scent, and color without the key ingredients is used. The placebo is composed of glucose, sucrose, lactose, corn starch, D-sorbitol, xanthan gum, and caramel color. With a 2-week follow-up, the subjects received a 14-day package per visit.

#### 2.4.3 Management of the drug

The clinical trial drug is kept in airtight containers at room temperature (21–30°C). The clinical trial drug is administered by the pharmacist in accordance with the investigator's prescription, and the pharmacist accurately records the clinical trial drug provided to the study subjects and management status. Since this is a double-blind clinical trial, study subjects are only identifiable through their identification number, and information about the prescribed and collected clinical trial drug should be appropriately recorded. Upon termination or completion of the clinical trial, the pharmacist must return the unused clinical trial drug to the sponsor, retain the proof of return, and the sponsor shall dispose of the returned clinical trial drug.

### 2.5 Outcomes

#### 2.5.1 Primary outcome

##### 2.5.1.1 Brief Fatigue Inventory (BFI)

The BFI is a tool developed for the fast and simple measurement of fatigue and is widely used in studies.^[19]^ It is composed of 9 questions, with 3 questions for the degree of CRF and 6 questions for the effects of CRF. Each question is scored from 0 to 10 points, where a higher score indicates severe symptoms; 1 to 3 point is mild, 4 to 6 point is moderate, and 7 to 10 point is severe. Measurements are taken at the baseline, 2 weeks, 4 weeks, 6 weeks, and 10 weeks after treatment (Table 1).

**Table 1:**
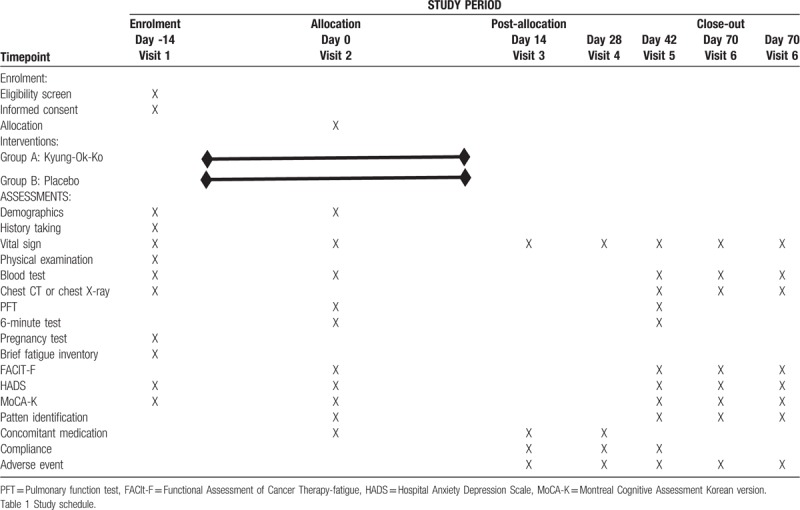
Trial schedule.

#### 2.5.2 Secondary outcomes

##### 2.5.2.1 Visual Analogue Fatigue Scale (VAFS)

The VAFS measures from 0 (not tired at all) to 10 (very tired) and classified as: Grade 1 or mild (1,2,3), Grade 2 or moderate (4,5,6), and Grade 3 or severe (7,8,9,10) using National Cancer Institute Common Toxicity Criteria version 4.03. Measurements are taken at the baseline, 2 weeks, 4 weeks, 6 weeks, and 10 weeks after treatment.

##### 2.5.2.2 Functional Assessment of Cancer Therapy (FACIT) Fatigue scale

The FACIT Fatigue scale is composed of 13 questions, and the cancer patient is required to give a score to the statement that best represents his or her weekly experience.^[20]^ The 13 questions of this measurement tool are based on the cancer patient's feelings and experience with fatigue, eating and sleeping with fatigue, activity status, and fatigue experienced in the past week. It is scored from 0 points for ‘Strongly disagree’ to 4 points for ‘Strongly agree’, where a higher score indicates a high degree of fatigue. Measurements are taken at the baseline, 2 weeks, 4 weeks, 6 weeks, and 10 weeks after treatment.

##### 2.5.2.3 Hospital Anxiety Depression Scale (HADS)

The HADS is an efficient screening tool generally used to assess the anxiety and depression of hospitalized cancer patients.^[21]^ It is composed of 2 subcategories, anxiety and depression, with a total of 14 questions, consisting of 7 questions for anxiety and 7 questions for depression. Each question is based on a 3-point scale, with a total of 21 points possible for each subcategory of anxiety and depression. A higher score indicates a high degree of anxiety and depression, where 0 to 7 points indicate no anxiety and depression, 8 to 10 points suspect anxiety and depression, and >11 points indicate anxiety and depression.^[21]^ Moreover, the total scores refer to a combination of anxiety and depression scores and classified as mild, ranging from 8 to 10, moderate from 11 to 14, and severe from 15 to 21 points.^[22]^ Measurements are taken at the baseline, 2 weeks, 4 weeks, 6 weeks, and 10 weeks after treatment.

##### 2.5.2.4 Montreal Cognitive Assessment Korean version (MoCA-K)

The Korean version of Montreal Cognitive Assessment is a tool developed to examine mild cognitive impairment.^[23]^ It consists of 30 points in total, consisting of short-term memory test (5 points), clock drawing for space-time function (3 points) and 3-dimensional block drawing tests (1 point), trail-making test B for performance (1 point), language fluency test (1 point) and 2-item verbal abstraction task (2 points), concentration maintenance test to test concentration (1 point), subtraction in order (3 points), digit span forward and backward (2 points), animal names (3 points) and following speaking complex phrases (2 points), and disorientation for time and place (6 points). If the total score is <22, mild cognitive impairment can be suspected. Moreover, mild cognitive impairment score ranges from 18 to 26 points, moderate from 10 to 17 points, and severe if <10 points. Measurements are taken at the baseline, 2 weeks, 4 weeks, 6 weeks, and 10 weeks after treatment.

##### 2.5.2.5 Korean pattern identification questionnaire

Qi Blood Yin Yang Deficiency Questionnaire is based on a 4-point Likert scale, composed of 15 questions.^[24]^ It was developed as a tool to assess chronic fatigue, and studies on its validity have been conducted.^[25]^ Measurements are taken at the baseline, 2 weeks, 4 weeks, 6 weeks, and 10 weeks after treatment.

##### 2.5.2.6 Inflammation markers

Many researchers have reported changes in the immune and inflammatory responses as a mechanism of CRF and inflammation-related cytokine studies, in relation to CRF, have been conducted.^[26–30]^ In this regard, we aimed to examine the inflammatory markers as one of the objective indicators of the efficacy of Kyung-Ok-Ko in improving fatigue. Inflammatory markers included C-reactive protein, erythrocyte sedimentation rate, and cytokines, such as interleukin (IL)-1, IL-6, tumor necrosis factor (TNF)-α, IL-1ra, IL-8, IL-10, and interferon (IFN)-γ. Measurements are taken at the baseline and 6 weeks after treatment.

### 2.6 Safety

AEs refer to undesirable, unintended signs, symptoms, or diseases that occur during clinical trials and do not necessarily have causal relationships with treatments used in the trial. The presence of AEs should be checked at every visit. The severity of AEs will be evaluated by Common Terminology Criteria for Adverse Events (CTCAE). An SAE refers to one of the following AEs occurring in a clinical study participant during a clinical trial:

(1) Death or danger to life;
(2) Hospitalization or extension of hospital stay due to the adverse event;
(3) Permanent or significant failure or degradation of function;
(4) Development of fetal malformations or abnormalities;
(5) Other medically important situations.

In the case of SAEs, the investigators should report to the sponsor very quickly (usually within 24 hour). This would ensure patient safety in the clinical trials and meet the reporting requirements of the Korean MFDS. In addition to reporting AEs to the sponsor, the investigator also reports to the Institutional Review Board (IRB). To assess the safety, laboratory tests determined the AST, ALT, alkaline phosphatase, total bilirubin, gamma glutamyl transferase, blood urea nitrogen, and creatinine. Measurements are taken at the baseline, 2 weeks, 4 weeks, 6 weeks, and 10 weeks after treatment.

### 2.7 Sample size

The sample size was estimated following a similar clinical research study because the exact same study design has not been employed before. The primary endpoint is the BFI, and the sample size is calculated based on the effect size of the primary endpoint. According to the previous literature,^[31]^ the mean and standard deviation of BFI were 3.4 ± 1.6 and 5.2 ± 1.9 for the experimental group and placebo group, respectively. Using a 0.05 significance level, 90% power, and allowing for a 10% dropout rate, a sample size of 50 participants was estimated, 25 in each group.

### 2.8 Recruitment

Among lung cancer patients receiving cancer treatment, patients who experienced CRF are recruited from the division of pulmonology and radiation oncology of the Kyung Hee University Medical Center. This clinical trial is to be conducted at K-CTC. The purpose of this study is explained to the subjects, and screening is conducted among those who signed the informed consent form.

### 2.9 Data management and monitoring

Data collection is conducted based on the approved protocol. The collected data is recorded in the case report. The quality of the study will be managed by regular monitoring. An independent monitoring supervisor would be assigned to contact and visit the researchers regularly, and thus, supervise the trial process. All investigators will be trained and will comply with GCP regulations. All documents related to the clinical trial will be archived by the principal investigator and the manager. Data will be stored in a separate place with security. The participants’ information will be maintained in storage for a period of 3 years after study completion. In case of a change in the protocol, it will be immediately reported to the IRB, and all investigators will be informed

### 2.10 Ethics

This trial has been authorized by the IRB of the Kyung Hee University Korean Medicine Hospital (KOMCIRB 2018-05-017-001). The protocol complies with both the Declaration of Helsinki and GCP Guidelines. Signed informed consent forms will be obtained from all eligible participants before enrollment.

This trial is registered with the Korean Clinical Trial Registry (KCT0003115).

### 2.11 Statistical methods

Statistical significance is based on the significance level 0.05 with a power of 0.8.

- ITT: Includes all subjects who were administered with the study drug or placebo at least once, and performed and returned the evaluation sheet for BFI.
- PP: Includes all subjects who were administered with >80% of the assigned study drug or placebo, and completed the evaluation sheet for at least 34 days (80% of a total of 42 days) of BFI.
- For all analyses, ITT analysis is the major analysis, and PP analysis is simultaneously performed to present the results together.

Continuous variables are presented as mean and standard deviation, and independent t test or Mann–Whitney *U* test is performed. Categorical variables are presented as frequency and ratio, and Chi-square test or Fisher's exact test (when the expected frequency is less than 5 at 25% of the total value) is performed.

Primary efficacy evaluation is the comparison of the mean of BFI score assessed at week 6 (42 days after administration) between the groups. The mean difference between the baseline and week 6 of VAFS, FACIT-F, MoCA-K, HADS and inflammation markers are to be compared. Independent *t* test is performed if normality test is satisfied, and Mann–Whitney *U* test is performed if the normality test is not satisfied. If a significant difference between groups is found for the baseline values, ANCOVA is performed. The missing value will be replaced by the last measured value of each subject, according to the Last Observation Carried Forward (LOCF) method.

## 3 Discussion

This study is the first research protocol to clarify the efficacy and safety of Kyung-Ok-Ko in CRF in lung cancer patients. Kyung-Ok-Ko is widely used in East-Asian countries, especially Korea, for recovery from fatigue and the reinvigoration of physical strength. In addition, Kyung-Ok-Ko is, clinically, the most frequently used drug to improve CRF. Kyung-ok-ko has been proven to have anti-inflammatory effects in a variety of illnesses.^[16–18]^ A study had demonstrated its effect in lung cancer, and the antifatigue effect has also been studied.^[12,13]^ However, a study to investigate the efficacy and safety of Kyung-Ok-Ko in CRF, which is not easily alleviated, and thus, affects the quality of life and prognosis of cancer patients, is yet to be conducted.

Most of the major causes of CRF are side effects of cancer therapy, and it is commonly found that the fatigue mechanism is associated with inflammation. It has been reported that inflammation occurs because the cytokines affecting the brain induce many neurological, physiological, and behavioral changes, causing fatigue.^[26]^ Observational research has found that fatigue is induced by the presence of inflammatory markers,^[27]^ and a pilot study found that fatigue was improved by blocking TNF-α in cancer patients who had experienced fatigue.^[28]^ Based on the previous studies, which have shown the anti-inflammatory effects of Kyung-Ok-Ko, this research was designed to investigate the effect of Kyung-Ok-Ko in the regulation of inflammatory cytokines to reveal the treatment mechanisms of CRF.

This study has the following strengths. First, we unified only lung cancer to examine the efficacy and safety of Kyung-Ok-Ko in fatigue. Second, the study could contribute to the identification of a mechanism in association with inflammation, which is considered as a fatigue mechanism, by observing cytokine level before and after Kyung-Ok-Ko prescription. Third, we developed a dark-brown placebo paste preparation. To achieve this, the researchers had several meetings and tested various prototypes to develop the placebo. Although it was difficult to match the unique fragrance and viscosity of the traditional Korean medicine, we finally made a similar placebo, which made it possible to “blind” the patients and the researcher. Fourth, the study protocol was established closely following the Guidelines for GCP and the Consolidated Standards of Reporting Trials (CONSORT).

In conclusion, the study is aimed to examine the efficacy and safety of Kyung-Ok-Ko in treating CRF in lung cancer patients and establish high-quality evidence for clinicians and patients who are willing to use Kyung-Ok-Ko for CRF.

## Author contributions

**Conceptualization:** BJL

**Data curation:** MK

**Investigation:** SHL

**Methodology:** MK, SHL

**Project administration:** MK, SHL

**Supervision:** BJL

**Writing – original draft:** KIK

**Writing – review & editing:** KIK, BJL
